# The Influence of Reverse Total Shoulder Arthroplasty Implant Design on Biomechanics

**DOI:** 10.1007/s12178-023-09820-8

**Published:** 2023-02-03

**Authors:** Charles J. Cogan, Jason C. Ho, Vahid Entezari, Joseph P. Iannotti, Eric T. Ricchetti

**Affiliations:** 1 Department of Orthopaedic Surgery, Orthopaedic and Rheumatologic Institute, Cleveland Clinic, Cleveland, OH USA; 2 Department of Orthopaedic Surgery, University of California San Francisco, San Francisco, CA USA

**Keywords:** Reverse total shoulder arthroplasty, Biomechanics, Glenoid offset, Humeral offset

## Abstract

**Purpose of Review:**

As reverse total shoulder arthroplasty indications have expanded and the incidence of its use has increased, developments in implant design have been a critical component of its success. The purpose of this review is to highlight the recent literature regarding the effect of implant design on reverse total shoulder arthroplasty biomechanics.

**Recent Findings:**

Implant design for reverse total shoulder arthroplasty has evolved considerably from the modern design developed by Paul Grammont. The Grammont design had a medialized center of rotation and distalized humerus resulting from a 155° humeral neck shaft angle. These changes intended to decrease the forces on the glenoid component, thereby decreasing the risk for implant loosening and improving the deltoid moment arm. However, these features also led to scapular notching. The Grammont design has been modified over the last 20 years to increase the lateral offset of the glenosphere and decrease the prosthetic humeral neck shaft angle to 135°. These changes were made to optimize functional range of motion while minimizing scapular notching and improving active external rotation strength. Lastly, the introduction of preoperative planning and patient-specific instrumentation has improved surgeon ability to accurately place implants and optimize impingement-free range of motion.

**Summary:**

Success and durability of the reverse total shoulder arthroplasty has been contingent upon changes in implant design, starting with the Grammont-style prosthesis. Current humeral and glenoid implant designs vary in parameters such as humeral and glenoid offset, humeral tray design, liner thickness, and neck-shaft angle. A better understanding of the biomechanical implications of these design parameters will allow us to optimize shoulder function and minimize implant-related complications after reverse total shoulder arthroplasty.

## Introduction

Shoulder arthroplasty has been a means of treating shoulder pain and dysfunction due to arthritis since the 1800s, with pioneers such as Themistocles Gluck and Jules Emile Péan performing the first total shoulder arthroplasties [1]. Charles Neer was among the first surgeons in the mid-twentieth century to develop the modern era of anatomic total shoulder arthroplasty for both proximal humerus fracture and shoulder arthritis [1]. He noted, however, that patients with severe rotator cuff disease had worse outcomes due to humeral head migration [2], and this led to the development of a more inherently stable, constrained device—the reverse total shoulder arthroplasty (RTSA) [3]. The goal of this review article is to highlight the influence of various RTSA implant design features on shoulder biomechanics.

## Reverse Total Shoulder Biomechanics

The central principal of the RTSA design is to reverse the articulation of the joint, such that the convex ball is positioned on the glenoid and the concave socket is placed on the proximal humerus. This change confers several important advantages for shoulder function, as well as a few disadvantages, in comparison to anatomic shoulder biomechanics.

### Advantages

**Fig. 1:**
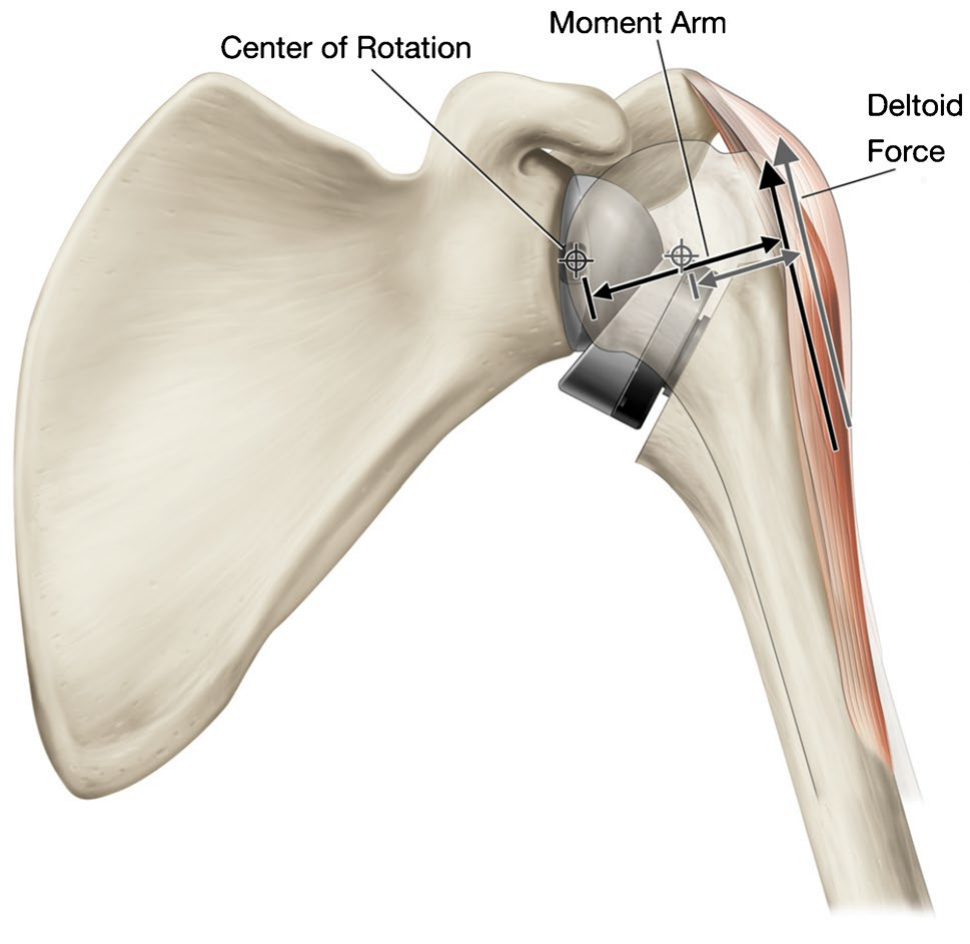
The medialization in center of rotation (COR) of the reverse total shoulder arthroplasty as compared to a native shoulder joint. The increase in deltoid moment arm and resultant force is demonstrated with medialization of the COR. With permission from Roche CP. Reverse shoulder arthroplasty biomechanics. JFMK. 2022;7:1–17 [4]

The first important biomechanical advantage of the Grammont-style RTSA is the medialization and distalization of the glenohumeral joint center of rotation (COR) (Fig. 1).

Early designs of the RTSA by Neer, Kessel, Reeves, and others maintained a COR that was lateral to the face of the glenoid. This lateralized COR increased shear forces at the glenoid bone-implant interface, leading to early failure due to scapula/glenoid fracture or component loosening [1, 3]. In 1991, Paul Grammont designed the Delta III prosthesis, which utilized a hemispherical glenosphere and a humeral component with 155° neck-shaft angle to medialize the COR and distalize the humerus. These changes provided multiple benefits, including decreasing the shear forces through the glenoid bone-implant interface, as well as increasing the deltoid abductor moment arm [4]. Furthermore, distalization of the humerus by increasing the neck shaft angle to 155° increased the deltoid tension across the glenohumeral joint, which improves stability of the prosthesis [5].

The second important advantage of the modern RTSA is an inherently stable prosthesis with a fixed fulcrum for rotation. In native shoulder biomechanics, the glenohumeral joint relies on a force coupling of the rotator cuff muscles to generate compressive forces that keep the humeral head centrally located and stable throughout range of motion [5–7]. In the setting of large to massive rotator cuff tears, this force couple becomes less effective, and the humeral head migrates superiorly. The end state of this process is anterosuperior escape of the humeral head, in which the humeral head migrates completely out of the contained coracoacromial arch. This can occur statically or actively with attempted forward elevation of the arm and results in loss of active shoulder elevation that is termed pseudoparalysis. This phenomenon is most commonly associated with a massive rotator cuff tear that includes most or all of the subscapularis tendon. To prevent humeral head migration, the RTSA is designed to have a more constrained articulation with a fixed fulcrum for humeral rotation, resulting from an equal radius of curvature (conformity) of the components and deeper articulation (constraint) of the joint. The constraint and conformity of the glenohumeral joint converts the deltoid moment arm to become more effective in forward flexion, but does not improve strength in rotation [6]. Rotational motion is still primarily dependent upon the remaining rotator cuff in RTSA, which also improves stability of the joint [8, 9].

### Disadvantages

**Fig. 2:**
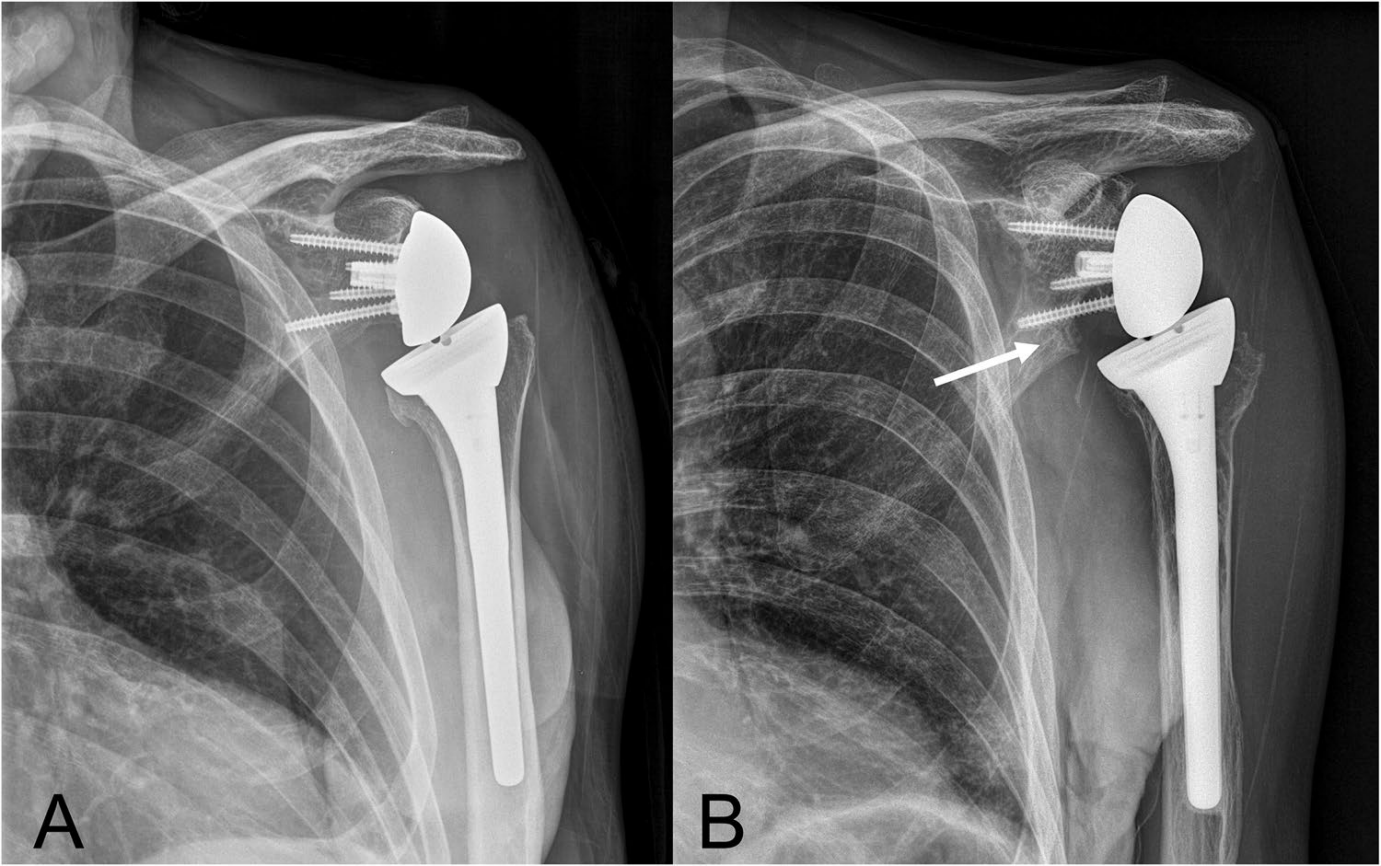
Demonstrates a Grammont design immediately postoperatively (**A**) as well as an 8-year follow-up radiograph where scapular notching is demonstrated (white arrow) (**B**)

While the aforementioned changes to the shoulder biomechanics have helped solve many issues of a rotator cuff deficient shoulder, there are still several functional disadvantages of the RTSA that have motivated the changes in implant design from the original Grammont prosthesis. The first disadvantage is related to the medialization of the COR and resulting medialization of the humerus. Medialization of the humerus shortens the length-tension relationship of the remaining rotator cuff muscles, impairing function for internal and external rotation of the shoulder [4, 5, 7]. Another important disadvantage to this medialized COR in combination with a 155° humeral neck shaft angle in the original Grammont design was the occurrence of scapular notching as a complication of RTSA (Fig. 2A, B).

With a hemispherical glenosphere and a humeral component with 155° neck-shaft angle, impingement occurs between the medial aspect of the humeral component and the scapular neck as the humerus is brought into adduction and/or rotation. This impingement is most pronounced in external rotation and triggers the development of scapular notching, which most likely represents a combination of bony impingement and polyethelene particulate-induced osteolysis as it progresses [10]. Over time scapular notching can lead to failure of glenoid baseplate fixation, decreased range of motion, and worse clinical outcomes [11, 12]. Furthermore, excessive polyethylene wear reduces the implant constraint and can lead to late instability. More modern RTSA designs have decreased bony impingement along the scapular neck through a combination of adding lateral offset to the glenoid component and utilizing a more varus neck-shaft angle of the humeral component in order to decrease the incidence of scapular notching and possibly improve rotational range of motion [13–15].

Another disadvantage to the RTSA design is the occurrence of acromial stress fracture as a complication. Distalization of the humerus relative to the native shoulder elongates the deltoid, which in combination with a greater deltoid moment arm increases load through the acromion [16]. This has been cited as a possible cause of acromial and scapular stress fracture after RTSA, which has an incidence of roughly 2–10% [16–19].

## Effect of Implant Design on Function

There are many important variations of implant design that affect shoulder function after RTSA. Understanding these differences is important for the surgeon and engineers who hope to optimize patient outcomes.

### Glenoid Mechanics

**Fig. 3:**
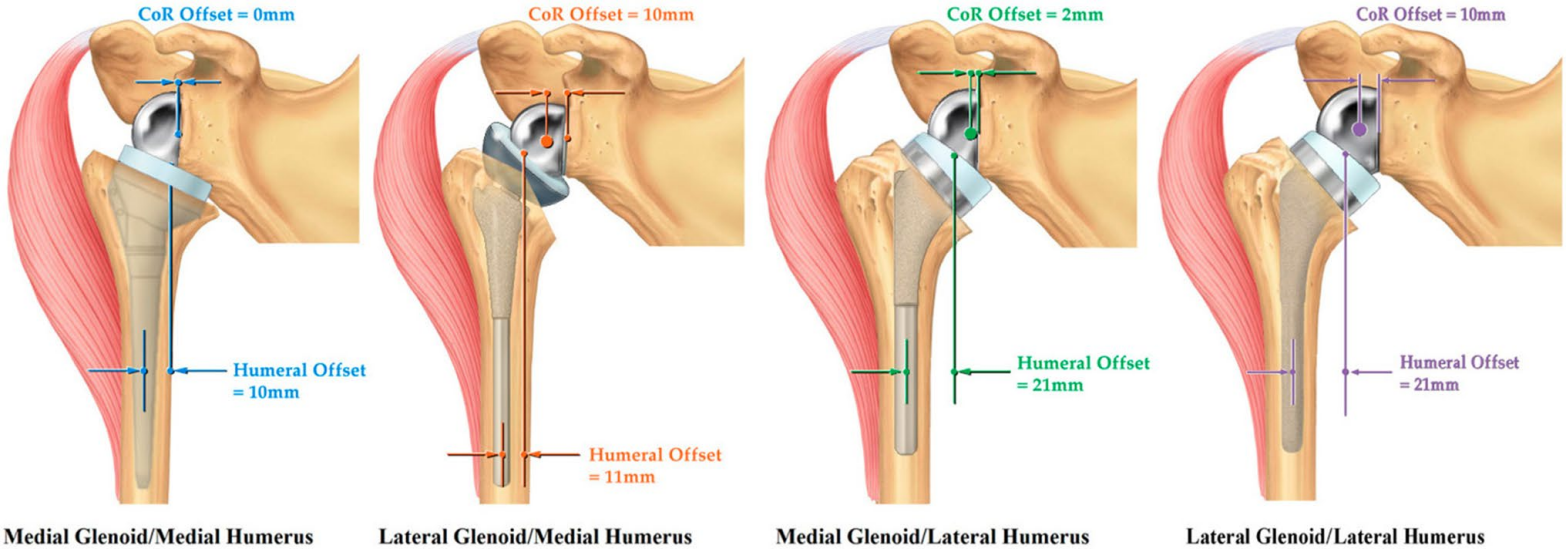
Reverse total shoulder arthroplasty design classification scheme. With permission from Roche CP. Reverse shoulder arthroplasty biomechanics. *JFMK*. 2022;7:1–17

There are multiple aspects of glenoid design which are important to consider. The first to discuss is glenoid offset, which is the amount of lateral projection of the glenoid component from the reamed glenoid surface, relative to its COR (Fig. 3).

Prosthetic lateralization of the COR through the glenoid component can be achieved through either the baseplate or the glenosphere. Some implants provide only one baseplate thickness, while others offer the opportunity to increase glenoid lateralization through multiple baseplate options. All current implant designs offer multiple sizing options for lateralization through the glenosphere based on two dimensions relative to a hemispherical design: glenosphere diameter and lateral offset. The implant diameter is measured as twice the radius of the glenosphere, while lateral offset is the additional thickness of the glenosphere beyond a hemisphere measured relative to the COR (Fig. 3). A hemispherical glenosphere would have 0 mm of lateral offset relative to its COR, for example, and would achieve lateralization only through the size of its diameter. This is termed a “Medial Glenoid” design (Fig. 3). Greater implant diameters alone result in more lateralization by increasing the radius of the glenosphere, and if used in combination with a more constrained socket will also have a greater jump distance, conferring more inherent joint stability by both soft tissue tension and increased constraint [4, 20, 21]. Increasing implant offset through the baseplate or glenosphere further lateralizes its COR, which has the advantage of restoring native rotator cuff moment arms, but it does decrease the deltoid moment arm and increases shear forces at the glenoid-implant interface [22]. Some authors have also reported that an overly lateralized COR may be a risk factor for acromial stress fractures. This was observed clinically in a study by Levy et al., where the lateralized glenoid implants had an acromial stress fracture rate of 10.2% [23]. The proposed mechanism of acromial stress fracture in this setting may be multifactorial. Excessive glenoid lateralization can increase strain along the base of the acromion as well as increase superior impingement between the greater tuberosity and acromion, which may be another cause of acromial stress fracture [24]. Implant offset also contributes to impingement-free range of motion, as each millimeter of offset correlates to 5° improved adduction prior to abutment of the humeral component on the inferior scapular neck [25].

Regarding glenoid design, one must also consider the eccentricity of the glenosphere. This feature can be utilized to shift the glenosphere inferiorly, allowing for overhang of the implant over the scapular neck and increased adduction before impingement. An alternative way to achieve this same effect with a centered, non-eccentric glenosphere is to place the baseplate inferiorly on the glenoid and in 10–15° of inferior tilt [26]. It is important to note, however, that inferior tilt can worsen impingement in an implant with a medialized glenoid and high neck-shaft angle (e.g., 155° neck-shaft angle and 0 mm lateralization).

Increasing the impingement-free range of motion after RTSA is an important goal for the surgeon to maximize functional range of motion. Impingement-free range of motion is the arc of motion between the humeral and glenoid components before the polyethylene impinges on the scapula or the greater tuberosity impinges on the acromion. Gutiérrez et al. studied this in a simulated computer model validated from their prior work in a Sawbones model, and they found lateral glenoid offset had the greatest effect on increasing impingement-free abduction, followed by inferior glenosphere placement, and inferior glenosphere tilt, while there was minimal impact with changes in the humeral neck shaft angle [15, 27]. This impingement in adduction, also known as adduction deficit, leads to scapular notching. In their study, the humeral neck shaft angle had the greatest impact on adduction deficit, with 170° leading to worse range of motion than 130° or 150°. After neck shaft angle, they found inferior glenoid position to have the second greatest effect in decreasing adduction deficit, followed by inferior tilt, lateralized COR, and glenosphere diameter [15]. It has also been suggested in biomechanical and computational studies that a lateralized COR improves rotational range of motion due to a greater arc of impingement-free motion [28, 29]. A recent multicenter trial that compared clinical outcomes of 455 patients with glenoid offset ranging from 0 to 8 mm demonstrated improvements in internal rotation for offset greater than 6 mm, but no difference in external rotation [30]. It is unclear why the theoretical advantages of improved external rotation would not translate into clinical outcomes. Some have proposed that it may be related to soft tissue constraints or rotator cuff deficiency limiting functional range of motion, as well as altered scapulothoracic biomechanics or implant orientation [31, 32]. For example, in the case of an uncorrected B-type glenoid, the baseplate would remain overly retroverted, which would limit the beneficial effect of lateral offset on external rotation range of motion.

### Humeral Mechanics

**Fig. 4:**
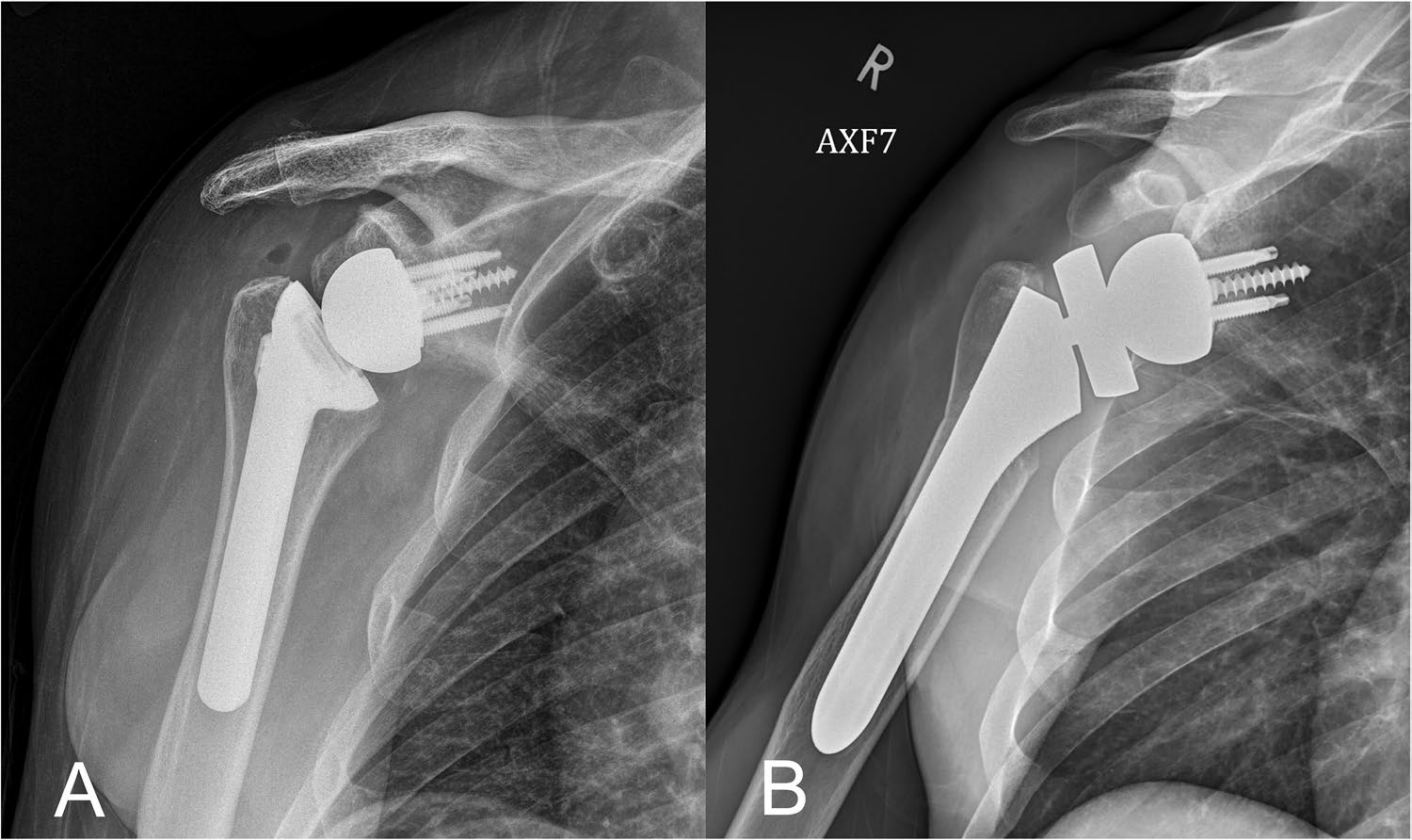
Reverse total shoulder arthroplasty with inlay humeral component (**A**) compared to an onlay humeral component (**B**). Increased humeral lateralization can be seen in to the onlay prosthesis, and both constructs have equal glenosphere size/offset and neck shaft angle

Various aspects of the humeral implant design will also affect the biomechanics of the RTSA, including humeral offset, humeral liner thickness and constraint, neck-shaft angle, and onlay versus inlay humeral design. Humeral offset is defined as the distance between the axis of the humeral implant shaft and the center of the polyethylene tray (Fig. 3). One of the advantages of increased humeral offset is the ability to re-tension the remaining rotator cuff and improve deltoid wrap without changing the implant COR [20]. Deltoid wrap can improve RTSA function due to added compressive stability, as well as improving the mechanical advantage of the posterior deltoid to aid in external rotation [20, 33]. Lateralization of the humeral component increases the deltoid wrap effect. The position of the humeral neck cut, the neck-shaft angle of the humeral implant, the thickness of the polyethylene humeral liner, and the use of an inlay or onlay humeral tray can all impact the amount of humeral offset. For instance, a 135° neck-shaft angle will increase offset more than a 155°. Furthermore, an onlay humeral tray will articulate with the glenosphere medial to the level of the humeral cut, which effectively lateralizes the humerus (Fig. 4). A more shallow humeral neck cut or using a thicker polyethylene liner in the humeral tray will have a similar effect, as they both add to length and, therefore, offset of the humeral side.

The inlay humeral tray design is theorized to restore a more anatomic position with regards to glenohumeral offset when used in combination with a lateralized glenosphere, which optimizes impingement-free range of motion [34]. In contrast, the benefits of an onlay humeral tray are more bone preservation as well as increased inherent stability through lateralization and distalization of the humeral implant [35]. Onlay designs have also allowed for ease of convertibility from anatomic TSA to RTSA at the time of revision in certain implant systems. A recent study comparing clinical outcomes of inlay and onlay humeral implants at minimum 2 years demonstrated improved external rotation and forward flexion in the onlay group, but greater rates of greater tuberosity and calcar resorption in the onlay group [34]. Interestingly, there was no difference in rates of scapular notching, which may be due to both groups utilizing lateral offset glenoids with a 135° neck shaft angle of the humeral components. In a computational study comparing implant design factors, Gutiérrez et al. found the neck-shaft angle to have the greatest effect in decreasing impingement [15]. There was significantly improved impingement-free range of motion for a neck shaft angle of 130° compared to 150° and 170°.

Humeral constraint is another design factor that affects biomechanical function. Humeral constraint is defined as the depth to width ratio of the humeral polyethylene liner [20], with most implants providing the option of more constrained or deeper liners, in addition to increasing liner thickness. Roche et al. found that each interval decrease in humeral constraint by 0.0125 decreased inferior impingement and increased range of motion by 4° [25]. Although decreasing humeral constraint will increase the impingement-free range of motion, there is a trade-off for less stability, as this will decrease the jump distance and increase risk of dislocation. The symmetry of the polyethylene liner also impacts impingement-free range of motion. Some manufacturers have asymmetric liners that alter the neck shaft angle from the humeral shell by increasing the thickness of the polyethylene inferiorly compared to superiorly. One biomechanical study by Permeswaran et al. demonstrated that anterior rotation of the asymmetric liner led to earlier impingement and posterior rotation allowed for more range of motion before impingement [36]. The difference between symmetric and asymmetric polyethylene liners in relation to impingement-free range of motion is not known.

## Classification System

In order to understand the biomechanical implications of different implant designs, Routman et al. developed a classification scheme based off of glenosphere and humeral implant offset [37]. The glenoid prosthesis is considered medialized (MG) if its COR is within 5 mm of the reamed glenoid face, and it is considered lateralized (LG) if its COR is greater than 5 mm from the reamed glenoid face. The humeral prosthesis is considered medialized (MH) if its offset from the center of the humeral tray to the humeral stem axis is 15 mm or less, and it is considered lateralized (LH) if this offset is greater than 15 mm. By combining these features, RTSA implants fall into one of the following categories: MG/MH, LG/MH, MG/LH, LG/LH (Fig. 3).

### MG/MH

The most common example of an MG/MH prosthesis is Paul Grammont’s Delta III design. The medialized COR maximizes the deltoid moment arm but shortens the rotator cuff muscles, with the end result being preserved forward flexion and abduction but compromised internal and external rotation [38]. Furthermore, these implants have historically had higher scapular notching rates due to the medialized design and valgus neck-shaft angle and often a repair of the subscapularis tendon has been recommended to improve stability [20, 39, 40].

### LG/MH

The LG/MH prostheses seek to provide better tensioning of the rotator cuff muscles, as this is believed to help with functional internal and external rotation. It is important to note that although denoted as LG, the COR is still relatively medial to the native shoulder COR. An LG/MH prosthesis typically has an inlay humeral component design used in combination with a lateralized glenosphere. Historically, some studies had shown relatively higher rates of glenoid implant loosening with early versions of the LG/MH design compared to MG implants [41, 42]. However, a change in implant design to improve fixation of the baseplate to the glenoid has eliminated this higher rate of loosening compared to MG designs, with durable outcomes noted at long-term follow-up [43]. Changes that have decreased failure rates specifically in an LG/MH prosthesis with 135° neck shaft angle of the humeral component include the use of locking 5.0-mm baseplate screws instead of non-locking 3.5-mm screws, options for more medialized COR in osteoporotic bone, and insertion of the baseplate with slight 10–15° inferior tilt [27, 41, 44]. Another technique that can lateralize the COR on the glenoid without increasing implant offset is the Bio-RSA, where humeral head autograft is used to lengthen the scapular neck [45]. This technique has shown good clinical outcomes [46–48], but is less commonly used than lateralizing through the glenoid baseplate or glenosphere.

### MG/LH

The MG/LH prostheses theoretically combine the benefits of a medialized COR (i.e., increased deltoid moment arm) with the benefits of a lateralized humerus (i.e., increased rotator cuff tension). An MG/LH prosthesis typically has an onlay humeral component design used in combination with a glenosphere that has 5 mm or less of lateral offset. One study comparing the amount of deltoid wrap and rotator cuff tensioning found that the MG/LH design had the most deltoid wrap and the least rotator cuff shortening compared to MG/MH and LG/MH designs [17]. It may be difficult or not possible to repair the subscapularis tendon in an implant with a LG/MH design, but it has been shown that this is not necessary for implant stability in an MG/LH prosthesis [40, 49]. The need for repair of the subscapularis tendon after RTSA remains inconclusive at this time [39, 40, 49, 50]. A meta-analysis by De Fine et al. demonstrated no difference between the repair and non-repair groups. Notably, the vast majority of the patients in this study had a lateralized design through either the glenoid or the humerus [51].

### LG/LH

An LG/LH prosthesis has not been commonly used in the clinical setting, as the combination of lateralization and distalization puts these implants at high risk for development of postoperative acromial stress fracture [52, 53].

## Preoperative Planning and Patient-Specific Instrumentation

One of the latest developments in RTSA implant design has been regarding patient-specific instrumentation (PSI) and preoperative planning that improve accuracy and fit of implant placement at the time of surgery [54, 55]. Custom-made or reusable PSI guides can be made by the implant company after planning on a preoperative three-dimensional CT scan to optimize the guide pin placement for the glenoid baseplate. In the absence of a PSI guide, the software for preoperative planning and templating of the procedure can also act as an intraoperative guide for implant position and selection [56, 57]. These planning software programs now also offer the ability to assess impingement-free range of motion with RTSA, to help determine the optimal combination of implant position, size, and amount of lateral offset.

Optimization of implant size and position to maximize impingement-free range of motion in the software may need modification in the operating room based upon soft tissue tension and ability to reduce the components. It is important to note that long-term benefit in clinical outcomes has not yet been established for PSI and planning over standard techniques in RTSA.

## Conclusion

Development of the RTSA has improved the success of shoulder arthroplasty for conditions such as rotator cuff tear arthropathy and proximal humerus fracture. Success of RTSA implants is based on the fundamental contributions from Grammont, whose design concept medializes the COR and allows the deltoid to effectively power shoulder flexion and abduction. The inherently stable implant design allows for rotation around a fixed fulcrum, which is important in a shoulder with a deficient rotator cuff. Scapular notching is a postoperative consequence of this medialized COR in combination with a more valgus neck-shaft angle of the humeral component and has been minimized by the development of more lateralized glenoid components in combination with humeral designs having lower neck-shaft angles. Differences in glenoid and humeral component design parameters affect the biomechanical function of the shoulder. Knowledge of how these differences affect function is important for improvement of implant designs and ultimately, patient outcomes.
